# Hierarchical level features based trainable segmentation for electron microscopy images

**DOI:** 10.1186/1475-925X-12-59

**Published:** 2013-06-28

**Authors:** Shuangling Wang, Guibao Cao, Benzheng Wei, Yilong Yin, Gongping Yang, Chunming Li

**Affiliations:** 1 School of Computer Science and Technology, Shandong University, Jinan 250101, China; 2 College of Science and Technology, Shandong University of Traditional Chinese Medicine, Jinan 250355, China; 3 Department of Radiology, University of Pennsylvania, Philadelphia, PA 19104, USA

**Keywords:** Machine learning, Biomedical image processing, Hierarchical level features, Random Forest, Membrane segmentation

## Abstract

**Background:**

The neuronal electron microscopy images segmentation is the basic and key step to efficiently build the 3D brain structure and connectivity for a better understanding of central neural system. However, due to the visual complex appearance of neuronal structures, it is challenging to automatically segment membranes from the EM images.

**Methods:**

In this paper, we present a fast, efficient segmentation method for neuronal EM images that utilizes hierarchical level features based on supervised learning. Hierarchical level features are designed by combining pixel and superpixel information to describe the EM image. For pixels in a superpixel have similar characteristics, only part of them is automatically selected and used to reduce information redundancy. To each selected pixel, 34 dimensional features are extracted by traditional way. Each superpixel itself is viewed as a unit to extract 35 dimensional features with statistical method. Also, 3 dimensional context level features among multi superpixels are extracted. Above three kinds of features are combined as a feature vector, namely, hierarchical level features to use for segmentation. Random forest is used as classifier and is trained with hierarchical level features to perform segmentation.

**Results:**

In small sample condition and with low-dimensional features, the effectiveness of our method is verified on the data set of ISBI2012 EM Segmentation Challenge, and its rand error, warping error and pixel error attain to 0.106308715, 0.001200104 and 0.079132453, respectively.

**Conclusions:**

Comparing to pixel level or superpixel level features, hierarchical level features have better discrimination ability and the proposed method is promising for membrane segmentation.

## Introduction

**Figure 1:**
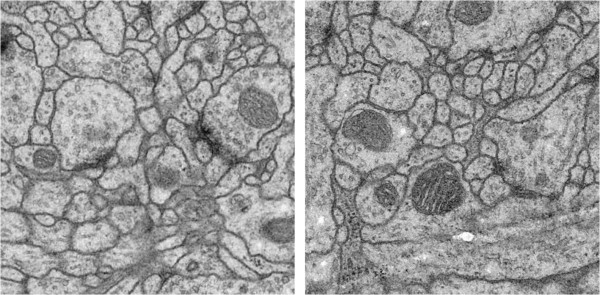
**Two slices of EM images.** Slices show us the complex cell textures and structures with largely varying shapes and topologies.

In order to get new insights into the functional structure of the brain, neuroanatomists need to reconstruct and mapping neural connections in 3D, which the key approach is 2D neuron image segmentation. Currently, electron microscopy (EM) is the main imaging tool which can provide sufficient resolution for studying connections at the neuron level. EM enables acquisition of huge datasets, which make tedious and time-consuming manual analysis simply infeasible. Therefore, accurately automatic neuronal segmentation is naturally proposed to resolve this problem. However, the intricate cell textures and structures with largely varying shapes and topologies, etc. [1] make the automatic segmentation of neuronal EM images very challenging. Figure 1 shows two slices of EM images.

In previous works of membrane segmentation in EM images, two trends deserve paying much more attention. One is that more and more supervised machine learning methods are used. The other is to find more discriminative feature for segmentation. As to feature extraction for membrane segmentation in EM images, related works can be divided into two categories, namely implicit methods and explicit ones.

Implicit methods usually deployed deep learning paradigm, in which raw pixel intensities are often directly used as the input to train artificial neural network (ANN) or its variants. This paradigm can learn features automatically and effectively. Authors in [2] sampled raw pixel intensities from fixed size rectangular window to train a multilayer Convolutional Neural Network (CNN) [3] to segment membranes. Neuron membranes were detected in [4] by using a serial ANN architecture combined with a feature vector composed of image intensities sampled from a special stencil neighborhood. In this framework, classifiers are trained in sequential order and each ANN is allowed to use the classification result of the prior one as a part of the input. In [5] multi-scale stencil windows and radon-like features are used to learn a series of discriminative models to detect neuron membranes in EM Images. A special deep ANN is adopted by authors [6] as a pixel classifier and the label of each pixel is predicted from raw pixel values in a square window centered on it. In this method, each window pixel is mapped to a neuron in the input layer, followed by a succession of convolutional and max-polling layers.

**Figure 2:**
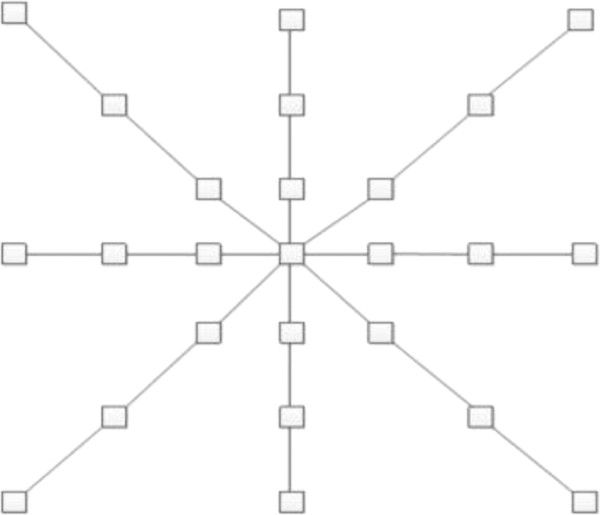
**Stencil neighborhood.** The stencil neighborhood is defined by a star shaped template with its 8 arms forking out every 45 degree.

As to explicit methods, image features are predefined before classifier is learned. In [7] the importance of context information for membrane segmentation is highlighted by using local context features computed from the hessian matrix to train a boosted classifier. The neighborhood is defined by a star shaped stencil with 8 arms (see Figure 2). By finding global dense correspondence between two sections, authors [8] exploited context information from the neighboring sections to reduce the ambiguities in neuronal segmentation of a section and extracted pixel level features to train a random forest classifier.

**Figure 3:**
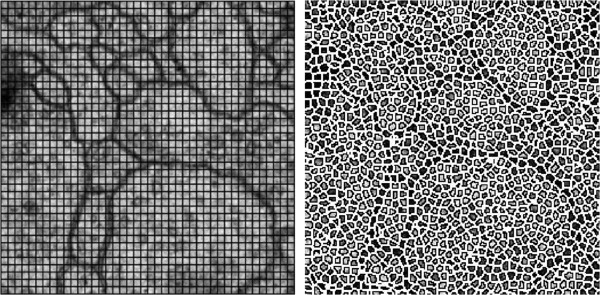
**Rectangular window-based neighborhood vs superpixel-based neighborhood.** Rectangular windows fail to correctly identify the boundaries of original images while superpixels preserve original image boundaries and enforce local consistency.

Although existed works achieve a reasonable segmentation result, they still have some challenges as following:

(1.) They tend to obtain context information sampled on rectangular (see Figure 3) or predefined stencil window (see Figure 2). Obviously such windows of fixed size or shape are inconsistent with the natural boundaries of images and one window may cover several distinct image regions. It will degrade the segmentation accuracy [9].

(2.) In order to get more discriminative context information, deep learning methods often adopt a large window, so that classifier training is computational intensive.

(3.) It is sensitive to noise and time consuming to implement segmentation only with pixel level features. Also, pixel level features could not describe local consistency characteristic of EM images.

Obtained by grouping nearby pixels, superpixel is a natural form of enforcing local consistency while respecting original image boundaries. Recent studies on superpixels showed that superpixels had superior performance to using rectangular image windows for localized image processing [9-11]. Moreover, superpixel, as a visual primitive, performs high efficiency for classification. For example, [11] gave a fully automated approach to segment mitochondria by using superpixel-based shape and local features. [10] performed automated nuclear segmentation by coupling superpixel context information with artificial neural networks. Although superpixel classifier is efficient, superpixel label is sensitive to the granularity of grouping. So superpixel classifier for EM images might be suitable for shaped and unconnected organelles segmentation.

Comparing to pixel, superpixel is a bigger granularity representation of the image. Pixels inside a superpixel have similar attributes, which is in reasonable agreement with the characteristic of local clustering of neuronal EM images. Superpixel-based features can be regarded as a kind of mid-level features, and pixel-based features as a kind of low level features. A natural idea is that superpixel level features may have different discriminative information from pixel level features for segmentation and the two kinds of features should be complementary at some degree. Superpixels naturally with the local information can be as a new formulation of adaptive windows neighborhood.

In the view of the above consideration, we define hierarchical level features which consist of pixel level features, superpixel level features and context level features among multi superpixels and random forest is used as classifier and is trained with hierarchical level features to perform segmentation. The effectiveness of our method is verified on the data set of ISBI2012 EM Segmentation Challenge.

## Methods

Framework based upon the proposed hierarchical level features and random forest for membrane segmentation is provided in the beginning of this section. Later, three important steps in this work, namely, preprocessing, trainable segmentation and postprocessing are presented. In the step of preprocessing, one newly proposed but widely used superpixel oversegmentation algorithm is introduced. In the stage of trainable segmentation, hierarchical level feature definition and extraction are given and Random Forest is used to implement segmentation.

**Figure 4:**
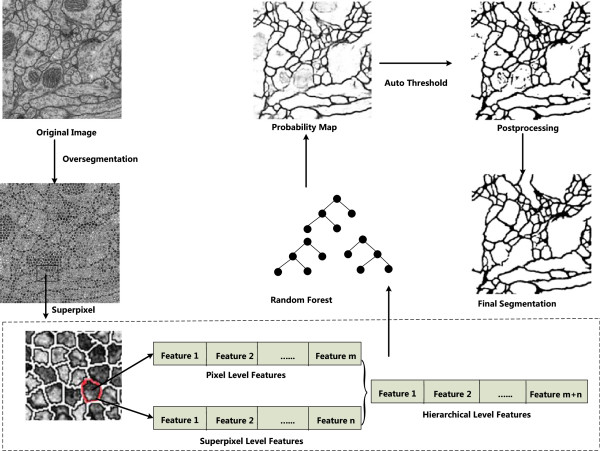
Overview of our method.

Figure 4 provides an overview of our method, and our method consists of the following three steps:

I. Preprocessing: image enhancement, superpixel oversegmentation

II. Trainable segmentation: sample selection, feature extraction and segmentation

III. Postprocessing

### Preprocessing

Before oversegmentation and feature extraction, images were preprocessed with histogram equalization and Gaussian filter to reduce noise, make the intensity more uniform and improve the contrast of the membranes.

### Superpixel oversegmentation

Image oversegmentation is a usual way to obtain superpixels, and superpixels demonstrate local clustering of images. Obtained by the nearby pixel grouping, superpixels might be a more natural and perceptually meaningful representation of reality [12]. It has been shown that using superpixels are advantageous because they can preserve natural image boundaries and reduce redundant information of the image data as well as enforce local consistency [11].

In this work, we apply Simple Linear Iterative Clustering (SLIC) [13] to generate superpixels, which is a variant of k-means algorithm. By integrating the local intensity and position information, SLIC divides an image into small patches. Superpixels obtained by SLIC are nearly uniform size and their boundaries are closely match true image boundaries. In addition, SLIC only need specify one parameter of the desired number of superpixels and has low computation complexity.

SLIC has with two important distinctions:

1. SLIC searches a limited region proportion to the superpixel size. $S=\sqrt{N/\text{k,}}$ where *s* is the grid interval, N is the number of pixels, and *k* is the desired number of superpixels. A superpixel is a region of approximate size *s* × *s*,the search for similar pixels is done in a region 2 *s* × 2 *s* around the superpixel center.

2. SLIC adopts a weighted distance measure *D,* as shown in Equations (1)-(3).

a. SLIC clusters pixels in the combined five dimensional color and image plane space to efficiently generate compact, nearly uniform superpixels.

(1) $$d_{s}=\sqrt{{\left(x_{j}-x_{i}\right)}^{2}+{\left(y_{j}-y_{i}\right)}^{2}}$$

(2) $$d_{c}=\sqrt{{\left(l_{j}-l_{i}\right)}^{2}+{\left(a_{j}-a_{i}\right)}^{2}+{\left(b_{j}-b_{i}\right)}^{2}}$$

(3) $$D=\sqrt{{\left(\frac{d_{c}}{N_{c}}\right)}^{2}+\left(\frac{d_{s}}{N_{s}}\right)}$$

where [*l a b*]^*T*^denotes color space, [*x y*]^*T*^ denotes the pixel’s position, [*l*_*k*_*,a*_*k*_*,b*_*k*_*,x*_*k,*_*y*_*k*_]^*T*^ denotes cluster center, *d*_s_ is space proximity, *d*_c_ is color proximity, and *N*_*c*_*, N*_*s*_ are the normalized factors.

### Trainable segmentation

#### Superpixel based automatic representative sample selection

**Figure 5:**
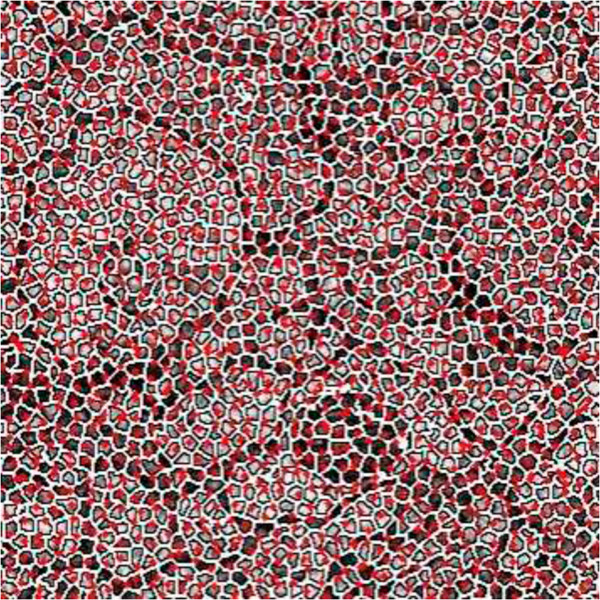
**Automatic training point selection.** Red points denotes selected points, and for illustration, one point corresponding to one superpixel.

For large volumes of EM data set, training pure pixel classifier is large time consuming and big storage space demanding. Pixels inside the same superpixel can be seen as homogenous, thus there are similarity and redundancy between pixels. So we apply superpixel based automated representative sample selection. Specifically, in each superpixel, we apply certain criterions (for example choosing points randomly) to select pixel samples to represent all the pixels inside this superpixel (see Figure 5). For simplicity, we just randomly select one point corresponding to one single superpixel in our work. Besides, this automated sample selection is robust to noise.

#### Hierarchical level features (HLFs)

##### Low level features extraction (pixel-based features extraction)

**Figure 6:**
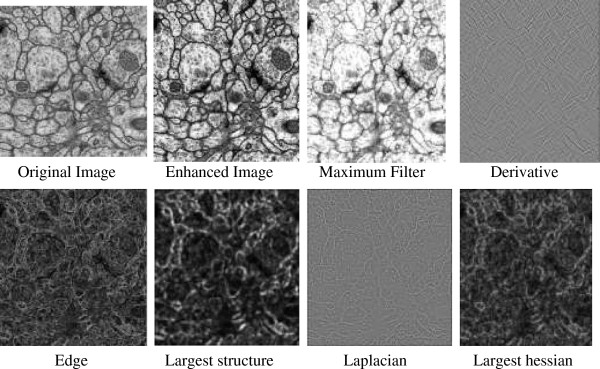
Selected examples of extracted features.

Most of pixel features are provided by Fiji with default parameters (http://fiji.sc/wiki/index.php/About). Fiji is a public software package for feature extraction. Our set of 34-dimensional pixel features is as following: (1) 6-dimensional filter values with 3*3 neighborhood: Mean, Median, Maximum, Minimum, Variance, Gaussian blur. (2) Gaussian filter values with Sigma 1.5, 2.0, 3.5, 4.0, 5.0, and 6.0, respectively. Sobel filter with 3*3 kernel size, Largest Hessian and Smallest Hessian, Difference of Gaussian, Linear Kuwahara, Laplacian, Largest Structure and Smallest Structure, Derivative. In addition, we incorporate 12-dimensional ray features [14] and 1-dimensional Radon-like feature [15], which are newly developed and proved to be informative for neuronal reconstruction. Selected features are depicted in Figure 6.

##### Mid-level features extraction (superpixel-based features extraction)

From the perspective of collection, we formulate one superpixel as a set of local nearby homogenous pixels. Then we extract superpixel level features with statistical methods, rather than simply make superpixel as an elementary unit to extract features. Advantages of extract the superpixel features with statistical methods are twofold: First, there are rich pixel level features, thereby using statistical methods for superpixel feature extraction greatly enriches the representations of superpixel. Second, many statistical methods such as mean, variance are simple and efficient. In this paper, we represent a superpixel by simply averaging various pixel level features, thus we can get 34-dimensional superpixel features. And superpixel entropy derived from superpixel intensity histogram is utilized as one dimension of superpixel features. In addition, we also use 3-dimensional context level features among multi superpixels, such as mean intensity, mean gradient, intensity variance of neighboring superpixels.

In total, we use 35-dimensional superpixel features and 3-dimensional superpixel context information. In fact, context level features among multi superpixels belong to superpixel level features.

##### Integrate low level features with mid-level features as the HLFs

To each pixel, we get its superpixel-based neighborhood, which is different from conventional rectangular window-based neighborhood. And the new method is not only naturally adaptive but also has more perceptual meaningful. In total, we utilize 72-dimensional features, in which there are 34-dimensional pixel features, 35-dimensional superpixel features and 3-dimensional superpixel context information.

#### Classification

After selecting a set of features, another important step is to select a good classifier. Random forest is a widely employed “ensemble learning” method for classification and regression proposed by Breiman. This method is robust and user-friendly as it has only two parameters and it can work efficiently on large data sets. Even in presence of many noisy features, Random Forest works well, so it is unnecessary to perform feature selection procedure. Considering all of the above, we adopt Random Forest to train the proposed HLFs.

Random forest is ensemble learning consists of tree predictors such that each tree depends on the values of a random vector sampled independently and with the same distribution for all trees in the forest [16]. Each tree is grown as follows: First, the best split at each node is selected from among a random subset of the predictor variables. Different variables are used at each split in different trees. Second, the training set used to grow each tree is a bootstrap sample of the observations, i.e., a sample of size *N* drawn with replacement from the original sample of *N* observations. Some observations are represented multiple times, while others are left out. The left-out observations are called “out-of-bag” and are used to estimate prediction error, eliminating the need for a test set or cross-validation. Third, each tree is grown to its large extent possible, and there is no pruning. The random forests final prediction is the mean prediction (regression) or class with maximum votes (classification). Breiman [16] showed that the prediction error converges to a limiting value as the number of trees tends to infinity.

### Postprocessing

As a post-processing procedure two steps are performed. The first step is auto-threshold methods provided by Fiji to membrane probability map returned by RF, for the purpose of improving membrane continuity. The second step is iteratively region removing, performed by a series of thresholding operations based on region properties such as Area, Euler Number, Solidity and Eccentricity.

## Experiments and results

### Description of the data set

The data we used in this paper provided by the organizers of the ISBI 2012 EM Segmentation Challenge (http://www.biomedicalimaging.org/2012/index.php). The data set consists of training data and testing data of the Drosophila first instar larva ventral nerve cord, which is provided in the form of EM stack.

**Figure 7:**
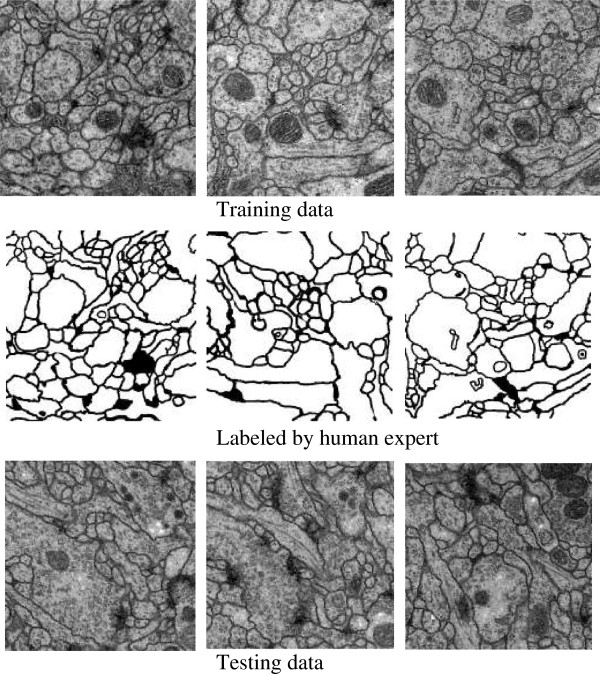
Selected slices of training data, corresponding label and test data.

The training data labeled by an expert human neuroanatomist is a set of 30 sections from a serial section Transmission Electron Microscopy (ssTEM) data set. The test data (ground truth unknown to the authors) consisting of 30 sections is another volume from the same Drosophila first instar larva ventral nerve cord. Some typical slices and their corresponding labels are depicted in Figure 7.

### Evaluation metrics

Segmentation result for challenge is evaluated through an automated online system; the system computes three error metrics in relation to the hidden ground truth: pixel error, warping error and the Rand error.

#### Pixel error

It is defined as 1 - the maximal F-score of pixel similarity or squared Euclidean distance between the original and the result labels [17].

#### Warping error

The warping error is segmentation metric that tolerates disagreements over boundary location, penalizes topological disagreements, and can be used directly as a cost function for learning boundary detection [17].

#### Rand error

The Rand error metric is based on the Rand index, defined as 1 - the maximal F-score of Rand index [18], a measure of similarity between two clusters or segmentations. It has a more intuitive interpretation, but completely disregards non-topological errors [6].

### Comparison experiments on different level features

We do experiment to compare the segmentation performance of pure pixel, pure superpixel and HLFs on the three metrics.

#### Experimental setting

We divide the public training data set into two parts: previous 15 sections as our training set, remaining 15 sections as our test set. In order to test the effect of superpixel size on classification accuracy, we set the parameter of superpixel number for each section of EM image ranging from 7500 to 11500. For simplicity, just one single pixel point is randomly selected corresponding to one superpixel.

#### Classification results

**Figure 8:**
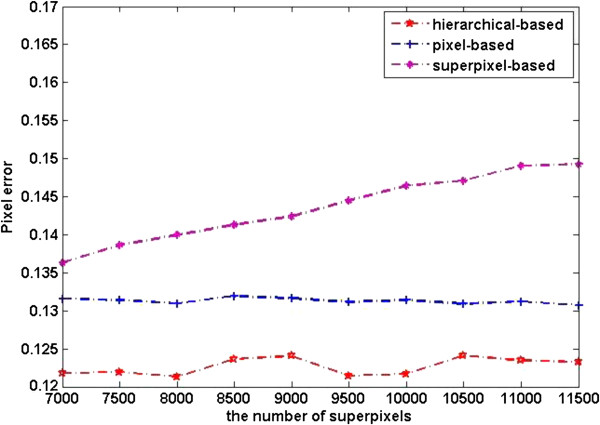
Pixel error of different level features.

**Figure 9:**
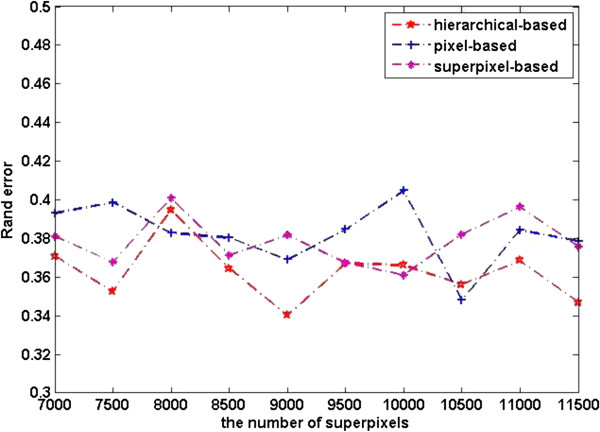
Rand error of different level features.

**Figure 10:**
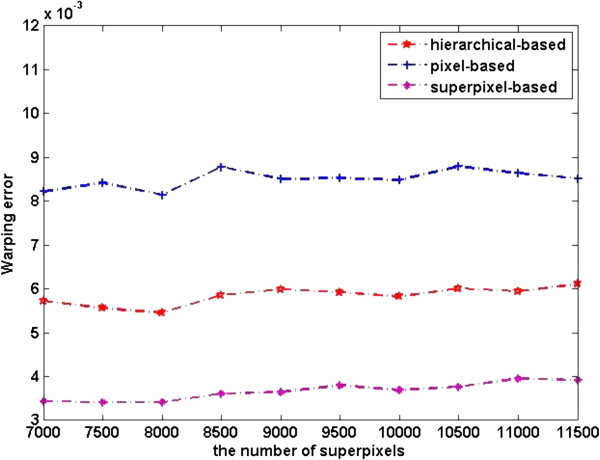
Warping error of different level features.

The quantitative segmentation results of the three kinds of level features are shown in Figures 8, 9, and 10.

The plots in Figure 8 show the pixel error on the dataset. We can see from the plots that pixel level features perform better than superpixel level ones. This can be explained as pixel level features have richer information than superpixel level ones, as a result the image can be represented better by pixel level features. Pixel error plot of superpixel level features shows an upward trend with the increasing of number of superpixels, this is because classification at superpixel level is sensitive to granularity of oversegmentation. However, superpixel based segmentation performs better in warping error, see Figure 10, this is due to superpixel inherently adheres well to the original image boundaries. Rand error plots in Figure 9 clearly demonstrate the substantial improvement in the segmentation by our method. We can draw a conclusion from Figures 9 and 10 that our method integrates the advantages of pixel level feature and superpixel level features.

### HLFs feed into different learning algorithms

Aim to validate the recognition performance of HLFs as well as to find the best suitable classifier, we conduct experiments for different learning algorithms supplied with HLFs. We choose several currently popular classifiers: Random Forest (RF), Support Vector Machine (SVM) [19], AdaBoost [20] as well as Linear classifier. All the classifiers are trained with default parameters. In this experiment, training set and testing set are similar to the previous experiment, and number of superpixel is 8000 for each image. Experiment results are showed in Table 1.

**Table 1:** Different learning algorithms supplied with HLFs

**Method**	**Rand error**	**Warping error**	**Pixel error**
HLFs-SVM	0.81862738	0.00482126	0.06570173
HLFs-AdaBoost	0.71340669	0.00370051	0.07302954
HLFs-Linear	0.41051885	0.00756352	0.13368690
HLFs-RF	0.29548843	0.00282007	0.09258893

From Table 1 we can see that HLFs-RF achieves the best segmentation performance in general. Due to all results are directly returned by classifier without any postprocessing and rand error is very sensitive to topological errors, rand error for all the four methods is not good enough. Training set is not sufficient, which is maybe another reason for the lower rand error. SVM performs well for segmentation in pixel error, while rand error is quite poor. We explain this as SVM works well for problem in small sample condition and balanced data. It can be stated that RF is the better classifier suitable for the membrane segmentation.

### Computational time

All the experiments are performed on a computer with a Core i7 950 3.06GHz processor, 24GB of RAM, and four GTX 580 graphics cards and implemented in MATLAB. For the HLFs-RF method, we computed the average computational time (in seconds) of one image with standard deviation in different stage of the proposed method. The statistical result is shown in Table 2.

**Table 2:** Average computational time for one image (in seconds) in different stage of HLFs-RF

**Stage of process**	**Oversegmentation (8000 superpixels) Mean time** **±** **std dev**	**Feature extraction (72 dimensions) Mean time** **±** **std dev**	**Classification Mean time** **±** **std dev**
Time	15.8797 ± 0.1339	148.1344 ± 3.0695	36.6854 ± 0.1339

From Table 2 we can see that the average computational time of one image with our method is about 201 seconds and it can basically satisfy actual application.

### The comparison experiment on the data set of ISBI2012

In this section, we compare our method with the two benchmark methods of ISBI2012 competition, namely Second Human Observer and Simple Thresholding, as well as two newly published methods of [8] and [21]. 1878 dimension features are used in Laptev’s method, while only 72 dimension features are used in our method. In addition, only part of pixels in each superpixel is used in our method, which means small sample condition.

Experimental results are shown in Table 3. Even with quite low dimensional features and in small sample condition, our method still shows better performance comparing to other four existed ones on the whole. It indicates that HLFs have more discriminative ability than pixel level features.

**Table 3:** Experimental results of several methods on the ISBI2012’s data set

**Method**	**Rand error**	**Warping error**	**Pixel error**	**Classifier input**
Second human observer	0.026546995	0.000344086	0.066553289	N/A
Simple thresholding	0.449664478	0.017141342	0.225194944	N/A
Laptev’s method	--	0.00062	0.079264809	1878 dimension features
Burget’s method	0.139038440	0.002641296	0.102285508	Local-level and Segment-level features
HLFs-RF	0.106308715	0.001200104	0.079132453	72 dimension features

“--”means unknown, [Laptev et al., MICCAI’2012] do not indicate the Rand error. N/A means not available.

## Conclusions and future work

This paper deals with the segmentation of neuronal structures in EM stacks based on supervised learning method. Considering the local clustering of neuronal EM images, we define and extract HLFs, which can be regarded as kind of more reasonable and natural feature representation, and use Random Forest as a classifier to implement segment of membranes with HLFs. Experimental validation demonstrates a promising performance of our method.

Future works will focus on two directions. One is to find more discriminative feature representation for superpixel. The other is to develop new classification techniques so that HLFs can be more fully utilized.

## Abbreviations

HLFs: Hierarchical Level Features; ISBI: IEEE International Symposium on Biomedical Imaging; EM: Electron Microscopy; ANN: Artificial Neural Network; CNN: Convolutional Neural Network; SLIC: Simple Linear Iterative Clustering; ssTEM: Serial section Transmission Electron Microscopy; RF: Random Forest; SVM: Support Vector Machine.

## Competing interests

The authors declare that they have no competing interests.

## Authors’ contributions

SW designed the proposed method and drafted the manuscript. Implementation and experiment results analysis were done by GC. BW helped in drafting the manuscript. YY supervised the project, contributed to discussion and analysis and participated in manuscript revisions. GY and CL participated in manuscript revisions and provided critical review that helped in improving the manuscript. All authors read and approved the final manuscript.

## References

[B1] Liu T Jurrus E Seyedhosseini M Ellisman M Tasdizen T Watershed merge tree classification for electron microscopy image segmentation Pattern Recognition (ICPR) 2012 133 137 21st Internarional Conference on, Tsukuba, Japan PMC4256108 25485310

[B2] Jain V Murray JF Roth F Turaga S Zhigulin V Briggman KL Helmstaedter MN Denk W Seung HS Supervised learning of image restoration with convolutional networks Computer vision, 2007 ICCV 2007 IEEE 11th international conference on Rio de Janeiro 2007 Brazil: IEEE 1 8

[B3] LeCun Y Bengio Y Convolutional networks for images, speech, and time series The handbook of brain theory and neural networks 1995 Cambridge, MA, USA: MIT Press 255 258

[B4] Jurrus E Paiva ARC Watanabe S Anderson JR Jones BW Whitaker RT Jorgensen EM Marc RE Tolga T Detection of neuron membranes in electron microscopy images using a serial neural network architecture Medical Image Analysis 2010 14 6 770 783 10.1016/j.media.2010.06.002 20598935 PMC2930201

[B5] Seyedhosseini M Kumar R Jurrus E Giuly R Ellisman M Pfister P Tasdizen T Detection of neuron membranes in electron microscopy images using multi-scale context and radon-like features Medical image computing and computer-assisted intervention– MICCAI. 2011 2011 Toronto, Canada: Springer 670 677 10.1007/978-3-642-23623-5_84 PMC3343875 22003676

[B6] Ciresan D Giusti A Schmidhuber J Deep neural networks segment neuronal membranes in electron microscopy images Advances in neural information processing systems 25. Lake Tahoe, Nevada, united states 2012 Cambridge, MA USA: MIT Press 2852 2860

[B7] Venkataraju KU Paiva A Jurrus E Tasdizen T Automatic markup of neural cell membranes using boosted decision stumps 2009 IEEE international symposium on biomedical imaging (ISBI). from nano to macro Boston, MA: IEEE Vols 1 and 2; 2009:1039–1042

[B8] Laptev D Vezhnevets A Dwivedi S Buhmann JM Anisotropic ssTEM image segmentation using dense correspondence across sections Medical image computing and computer-assisted intervention-MICCAI, 2012 2012 Nice, France: Springer 323 330 10.1007/978-3-642-33415-3_40 23285567

[B9] Kim D Oh SM Rehg JM Traversability classification for ugv navigation: a comparison of patch and superpixel representations Intelligent robots and systems, 2007I ROS 2007 2007 San Diego, California USA: EEE/RSJ International Conference 3166 3173

[B10] Ahmad AH Fuhai L Liang G Zhiyong W Michael JT Yehia M Stephen TC W Suzuki K, Wang F, Shen D, Yan P Automated nuclear segmentation of coherent anti-stokes Raman scattering microscopy images by coupling superpixel context information with artificial neural networks Machine learning in medical imaging, Volume 7009 2011 Berlin Heidelberg: Springer 317 325

[B11] Aurélien L Kevin S Radhakrishna A Vincent L Pascal F A fully automated approach to segmentation of irregularly shaped cellular structures in EM images Medical image computing and computer-assisted intervention – MICCAI, Volume 6362 2010 Berlin Heidelberg: Springer 463 471 10.1007/978-3-642-15745-5_57 20879348

[B12] Wang J Wang X VCells: simple and efficient superpixels using edge-weighted centroidal voronoi tessellations Pattern Analysis and Machine Intelligence, IEEE Transactions on (TPAMI) 2012 34 6 1241 1247 10.1109/TPAMI.2012.47 22331852

[B13] Achanta R Shaji A Smith K Lucchi A Fua P Susstrunk S SLIC superpixels compared to state-of-the-Art superpixel methods Pattern analysis and machine Intelligence, IEEE Transactions on (TPAMI) 2012 34 11 2274 2282 10.1109/TPAMI.2012.120 22641706

[B14] Smith K Carleton A Lepetit V Fast ray features for learning irregular shapes Computer vision(ICCV), 2009 IEEE 12th international conference 2009 Kyoto, Japan: IEEE 397 404

[B15] Kumar R Vázquez-Reina A Pfister H Radon-like features and their application to connectomics Computer Vision and Pattern Recognition Workshops (CVPRW), 2010 IEEE Computer Society Conference on 2010 San Francisco, California, USA: IEEE 186 193

[B16] Breiman L Random forests Machine Learning 2001 45 1 5 32 10.1023/A:1010933404324

[B17] Jain V Bollmann B Richardson M Berger DR Helmstaedter MN Briggman KL Denk W Bowden JB Mendenhall JM Abraham WC Harris KM Kasthuri N Hayworth KJ Schalek R Tapia JC Lichtman JW Seung HS Boundary learning by optimization with topological constraints Computer vision and pattern recognition (CVPR), IEEE conference on 2010 San Francisco, California: IEEE 2488 2495

[B18] Rand WM Objective criteria for the evaluation of clustering methods J Am Stat Assoc 1971 66 336 846 850 10.1080/01621459.1971.10482356

[B19] Cortes C Vapnik V Support vector machine Machine learning 1995 20 3 273 297

[B20] Yoav F Schapire RE A decision-theoretic generalization of on-line learning and an application to boosting Journal of Computer and System Sciences 1997 55 1 119 139 ISSN 0022–0000, 10.1006/jcss.1997.1504 10.1006/jcss.1997.1504

[B21] Burget R Uher V Masek J Trainable segmentation based on local-level and segment-level feature extraction 2012 IEEE international symposium on biomedical imaging (ISBI). from nano to macro 2012 Barcelona, Spain: IEEE

